# Fighting hospital sepsis

**DOI:** 10.1186/cc11993

**Published:** 2013-03-19

**Authors:** AR Calini, S Vesconi, R Fumagalli, S Marchesi, L Ghezzi, G Monti

**Affiliations:** 1 Ospedale Niguarda Ca' Granda, Milan, Italy

## Introduction

Sepsis accounts for a very high mortality. The Surviving Sepsis Campaign recommends a first 6 hours resuscitative bundle to improve patient outcome. Despite this, the bundle is poorly performed because of several organizational and cultural barriers. In recognition of this, we guess that an Educational and Organizational Intervention out of the ICUs could impact on septic patient outcome. In order to test our hypothesis we carried out, in 12 hospitals, a pre-intervention survey of the human and organizational resources (HOR) available in the management of septic patients. The aim is to seek any barrier potentially affecting correct Guidelines implementation.

## Methods

Thirty-nine medical wards (MW) and 12 emergency departments (ED) were enrolled. Every unit was asked to fill in a pre-agreed HOR Checklist focused on the main requirements suggested by the Guidelines.

## Results

Analysing the human resources available, we see that the bed-to-doctor ratio significantly (*P <*0.01) increases from the day to the night shift: from 6 to 43 beds per doctor on the MW (median). Otherwise, the ED staff remains roughly the same: from 3.5 to 2.5 doctors on duty (median). The analysis of the organizational tools (Table 1) points out a low percentage of hospitals having: a Diagnostic and Therapeutic Protocol for sepsis management (8.3%), some Hospital Empirical Antibiotic Therapy Guidelines (0%) and an Infective Source Eradication Protocol (8.3%). Moreover, just 25% of hospitals involve an infectious diseases expert in every case of severe sepsis or septic shock.

**Table 1:** 

Resource	Availability (%)
Diagnostic and therapeutic protocol for septic patient management	8.3
Use of early warning score for diagnosis and management	8.3
Sepsis team	25
Microbiology laboratory: open 7 days a week	66.6
Lactate dosage: 24 hours a day availability	83.3
Central venous catheter insertion (CVC) available 24 hours a day	91.6
Hospital empirical antibiotic therapy guidelines	0
Infectious diseases team advice in any case of severe sepsis septic shock	25
Infective source eradication	
Infective source eradication protocols	8.3
Intervention radiology available 24 hours a day	50
Operating room available 24 hours a day	58.3
Specific infection management protocol (that is, CVC infection)	58.3
Imaging reporting service available 7 days a week	41.6

## Conclusion

We guess that the poor availability of HOR showed by the hospitals could have a role in the Guidelines implementation and in the patient's outcome. Only a comparison between these results and data collected from a Clinical Checklist, focused on sepsis bundle compliance, and from a patient's outcome summary could confirm our hypothesis. This is the aim for our next part of the study.
